# Live attenuated virus vaccine protects against SARS-CoV-2 variants of concern B.1.1.7 (Alpha) and B.1.351 (Beta)

**DOI:** 10.1126/sciadv.abk0172

**Published:** 2021-12-01

**Authors:** Jakob Trimpert, Julia M. Adler, Kathrin Eschke, Azza Abdelgawad, Theresa C. Firsching, Nadine Ebert, Tran Thi Nhu Thao, Achim D. Gruber, Volker Thiel, Nikolaus Osterrieder, Dusan Kunec

**Affiliations:** 1 Institut für Virologie, Freie Universität Berlin, Berlin, Germany.; 2 Department of Infectious Diseases and Respiratory Medicine, Charité, Universitätsmedizin Berlin, Berlin, Germany.; 3 Institut für Tierpathologie, Freie Universität Berlin, Berlin, Germany.; 4 Institute of Virology and Immunology, Bern and Mittelhäusern, Switzerland.; 5 Department of Infectious Diseases and Pathobiology, Vetsuisse Faculty, University of Bern, Bern, Switzerland.; 6 Graduate School for Biomedical Science, University of Bern, Bern, Switzerland.; 7 Department of Infectious Diseases and Public Health, Jockey Club College of Veterinary Medicine and Life Sciences, City University of Hong Kong, Kowloon, Hong Kong.

## Abstract

Vaccines are instrumental and indispensable in the fight against the COVID-19 pandemic. Several recent SARS-CoV-2 variants are more transmissible and evade infection- or vaccine-induced protection. We constructed live attenuated vaccine candidates by large-scale recoding of the SARS-CoV-2 genome and showed that the lead candidate, designated sCPD9, protects Syrian hamsters from a challenge with ancestral virus. Here, we assessed immunogenicity and protective efficacy of sCPD9 in the Roborovski dwarf hamster, a nontransgenic rodent species that is highly susceptible to SARS-CoV-2 and severe COVID-19–like disease. We show that a single intranasal vaccination with sCPD9 elicited strong cross-neutralizing antibody responses against four current SARS-CoV-2 variants of concern, B.1.1.7 (Alpha), B.1.351 (Beta), B.1.1.28.1 (Gamma), and B.1.617.2 (Delta). The sCPD9 vaccine offered complete protection from COVID-19–like disease caused by the ancestral SARS-CoV-2 variant B.1 and the two variants of concern B.1.1.7 and B.1.351.

## Teaser

A single intranasal vaccination with live attenuated virus provides protection against VOC B.1.1.7 (Alpha) and B.1.351 (Beta).

## INTRODUCTION

The devastation wrought by the coronavirus disease 2019 (COVID-19) pandemic highlighted the urgent need for effective vaccines against severe acute respiratory syndrome coronavirus 2 (SARS-CoV-2). Vaccine development began immediately after the discovery of the virus and proceeded at an unprecedented pace and scale (*1*). Within less than a year from the onset of the pandemic, the concentrated efforts were crowned with the first vaccine authorizations (*2*, *3*). Shortly thereafter, vaccination campaigns began in many countries, focusing primarily on high-risk groups, such as the elderly and health care workers. Until now, mass vaccination campaigns confirmed that the licensed vaccines are highly effective in preventing symptomatic and asymptomatic SARS-CoV-2 infections, COVID-19–related hospitalizations, and deaths (*4*, *5*). To date, several vaccines have received emergency or full approval for use in many different countries (*1*, *6*), and additional vaccine candidates are in final phase 3 clinical trials (*1*, *6*). Although vaccine rollouts have been successful, primarily in many wealthy countries, vaccine supply remains scarce, and policymakers in most countries must prioritize vaccination programs to achieve the greatest public health benefit. To reduce the immense social and economic burden and ultimately stop the pandemic, it will be necessary to develop, manufacture, and administer billions of safe, effective, and affordable vaccine doses to a large proportion of the world’s population, likely on a recurring basis.

SARS-CoV-2, positioned at the opposite end of the incipient arms race, is rapidly evolving (*7*–*9*). Benefiting from its global presence, the virus continues to adapt to its new host and to infection- or vaccine-induced immunity. During the course of the pandemic, a number of genetic variants have emerged (*7*–*9*). Variants that exhibit increased infectivity, cause greater morbidity and mortality, or have the ability to evade infection- or vaccine-induced immunity pose an increased threat to public health. The World Health Organization (WHO) and other national health agencies have independently established classification systems that categorize emerging variants as variants of interest (VOIs), variants under investigation (VUIs), or variants of concern (VOCs) based on their risk to public health (Table 1) (*10*–*14*). In addition, to simplify communication with the public, the WHO recommends that VOIs and VOCs should also be labeled using the letters of the Greek alphabet. As of 12 August 2021, viruses belonging to lineages B.1.1.7 (Alpha), B.1.351 (Beta), B.1.1.28.1 (Gamma), and B.1.617.2 (Delta) are classified by several health agencies as VOCs (Table 1). In countries where they emerged, these variants rapidly supplanted the preexisting variants and started to spread globally.

**Table 1.** Classification of circulating genetic variants of SARS-CoV-2 that currently pose the greatest threat to public health (as of 10 August 2021). WHO, World Health Organization; CDC, U.S. Centers for Disease Control and Prevention; ECDC, European Centre for Disease Prevention and Control; PHE, Public Health England; VOC, variant of concern; VOI, variant of interest; VUI, variant under investigation.

**Lineage**	**Label**	**First detected in**	**Earliest samples**	**WHO**	**CDC**	**ECDC**	**PHE**
B.1.1.7	Alpha	United Kingdom	September 2020	VOC	VOC	VOC	VOC
B.1.1.7 + E484K	Alpha	United Kingdom	February 2021			VOC	VOC
B.1.351	Beta	South Africa	May 2020	VOC	VOC	VOC	VOC
B.1.1.28.1 (P.1)	Gamma	Brazil	November 2020	VOC	VOC	VOC	VOC
B.1.1.28.2 (P.2)	Zeta	Brazil	April 2020				VUI
B.1.1.28.3 (P.3)	Theta	Philippines	January 2021			VOI	VUI
B.1.617.1	Kappa	India	October 2020	VOI	VOI	VOI	VUI
B.1.617.2	Delta	India	October 2020	VOC	VOC	VOC	VOC
B.1.617.3		India	October 2020		VOI		VUI
B.1.427/B.1.429	Epsilon	USA	March 2020		VOI		
B.1.525	Eta	Nigeria	December 2020	VOI	VOI	VOI	VUI
B.1.526	Iota	USA	November 2020	VOI	VOI		
B.1.620		Lithuania	February 2021			VOI	
B.1.621		Colombia	January 2021			VOI	VUI
B.1.1.318		United Kingdom	February 2021				VUI
A.23.1 + E484K		United Kingdom	February 2021				VUI
AV.1		United Kingdom	May 2021				VUI
C.36.3		Thailand/Egypt	May 2021				VUI
C.37	Lambda	Peru	December 2020	VOI		VOI	VUI

The B.1.1.7 variant, first detected in the United Kingdom in December 2020, is 50 to 100% more transmissible and possibly also more lethal than earlier variants but shows no tendency to evade immunity induced by infection or vaccination (*9*, *15*, *16*). The B.1.1.7 variant has been detected in 132 countries and rapidly became the dominant variant in Europe and the United States. The B.1.351 variant, first detected in South Africa in May 2020, is not only more transmissible but also capable of reinfecting individuals and of breaking through vaccine protection (*16*–*19*). The B.1.1.28.1 variant, better known as P.1, is similar to B.1.351 in that both share some important mutations in the spike glycoprotein (E484K, K417N/T, and N501Y). B.1.1.28.1 emerged in late 2020 in Manaus, Brazil (*8*). Similar to the B.1.351 variant, it can cause reinfection because it can bypass immunity developed after infection with other virus variants (*8*, *19*). It is estimated that B.1.1.28.1 is 40 to 140% more transmissible, more pathogenic, and 10 to 80% more lethal than other variants (*8*). Most recently, on 7 May 2021, the WHO reclassified the B.1.617.2 variant, first detected in India, as a VOC due to its high transmissibility (*11*). As of August 2021, B.1.617.2 has largely outcompeted B.1.1.7 and is now the predominant variant in Europe and the United States. According to the WHO, B.1.617.2 is the most dangerous strain worldwide, and it has attracted considerable attention for its ability to evade infection- and vaccine-mediated protection (*20*).

## RESULTS

We constructed a series of live attenuated SARS-CoV-2 vaccine candidates by large-scale recoding of the SARS-CoV-2 genome (*21*). The SARS-CoV-2 genome was modified by codon pair deoptimization (CPD), an approach that has resulted in rapid and efficient attenuation of a variety of RNA (*22*, *23*) and DNA viruses (*24*, *25*). CPD is based on the observation that some codon pairs occur in protein-coding sequences significantly less or more frequently than would be statistically expected (*26*). In CPD, viral genes are recoded to contain an increased number of codon pairs that are statistically underrepresented (suboptimal) in the host and are designed to lead to attenuation of the recoded viruses (*22*–*24*). In the process of CPD recoding, the positions of synonymous codons in the recoded sequence are exchanged in such a way that the frequency of underrepresented codon pairs increases. Because CPD exchanges only the positions of synonymous codons in recoded sequences, the recoded, live attenuated virus contains exactly the same antigens as the pathogenic parent. The introduced genetic changes reduce protein production from the recoded genes and also the replication fitness of the mutant virus (*23*). The conserved antigenic identity and the remaining replicative potential enable the recoded attenuated virus to fully engage the immune system of the host and provoke strong immune responses (*22*–*24*).

**Fig. 1.**
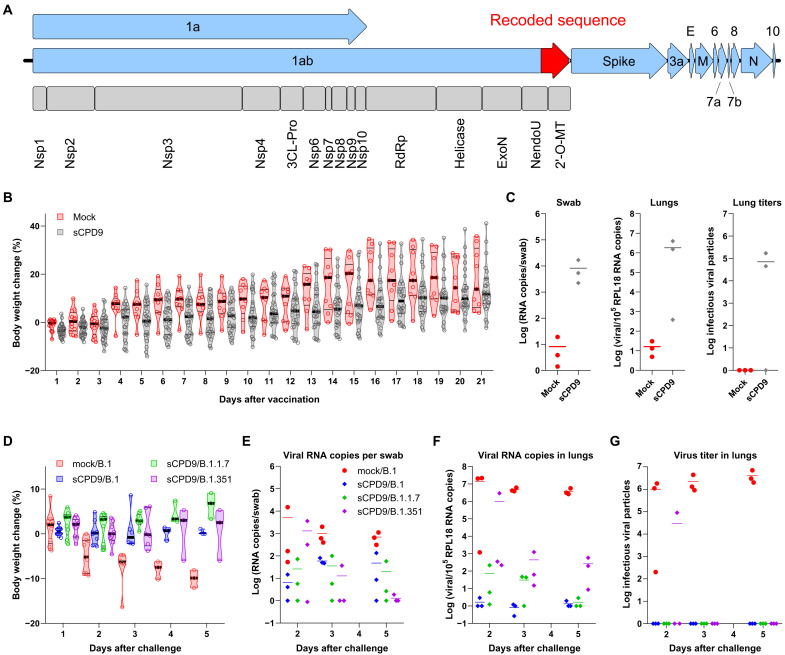
Live attenuated virus vaccine candidate sCPD9s is strongly attenuated and protects Roborovski dwarf hamsters from challenge with B.1, B.1.1.7, and B.1.351 viruses. (**A**) Schematic representation of the genome of the live attenuated virus sCPD9. The SARS-CoV-2 genome is a single-stranded, positive-sense mRNA molecule of approximately 30,000 nucleotides (nt), which encodes 11 canonical open reading frames (ORFs; blue arrows). After infection, ORF 1a/1ab is directly translated and cleaved into 15 proteins that constitute the replication-transcription complex. The genome of the sCPD9 vaccine candidate contains a 1,146-nt-long, recoded, codon pair–deoptimized sequence (red segment of the ORF 1ab). The recoded sequence is located near the 3′ end of ORF 1ab and encodes the nonstructural proteins endoribonuclease and 2′O-ribose methyltransferase (2′-O-MT). Nsp, nonstructural protein; 3CL-Pro, 3C-like proteinase; RdRp, RNA-dependent RNA polymerase; ExoN, 3′-to-5′ exoribonuclease; NendoU, endoribonuclease. (**B**) Body weight change of Roborovski dwarf hamsters after vaccination. The thick black lines show median, and the thin lines show the quartiles. (**C**) Viral load in the upper (oropharyngeal swab) and lower (lungs) respiratory tract and the number of infectious virus particles detected in 50 mg of lung tissue (lung titers) on day 3 after vaccination. (**D**) Body weight change of Roborovski dwarf hamsters after challenge with pathogenic B.1, B.1.1.7, or B.1.351 viruses. (**E** and **F**) Viral load in the upper (E) and lower (F) respiratory tracts of animals on days 2, 3, 5, and 5 after challenge. (**G**) Number of infectious virus particles detected in 50 mg of lung tissue on days 2, 3, and 5 after challenge. (B) and (C) were adapted from (*21*).

In our previous study, we have evaluated live attenuated virus candidates and have shown that some of them are strongly attenuated, induce strong immune responses, and protect Syrian hamsters from a challenge with ancestral wild-type (WT) SARS-CoV-2 (*21*). In that study, we also showed that the lead live attenuated virus candidate sCPD9 (Fig. 1A) is strongly attenuated in the Roborovski dwarf hamster (*Phodopus roborovskii*), which is highly susceptible to severe COVID-19–like disease (*27*). Despite the high susceptibility of this hamster species, sCPD9 did not cause clinical disease or considerable lung pathology (*21*).

The sCPD9 virus is also highly attenuated in vitro, where it grows to 100-fold lower titers than WT virus under normal conditions on Vero E6 cells (*21*). Nonetheless, it is possible to produce virus stocks with high titers (1 × 10^7^ infectious virus particles/ml) using the procedure described in Materials and Methods. In the current study, we assessed immunogenicity and protective efficacy of the sCPD9 virus against the ancestral B.1 and two VOCs, B.1.1.7 and B.1.351.

### Vaccination

Hamsters were randomly assigned to two groups of 12 and 30 animals. On day 0, 12 hamsters were mock-vaccinated and 30 hamsters were vaccinated with 1 × 10^5^ focus-forming units (FFUs) of the sCPD9 virus by intranasal instillation. On day 21, hamsters were challenged with 1 × 10^5^ plaque-forming units (PFUs) of the variants B.1, B.1.1.7, or B.1.351. Body weights and clinical signs were recorded daily for the duration of the experiment (26 days). Three animals of each group per time point were euthanized on day 3 after vaccination and on days 2, 3, and 5 after challenge to determine the degree of virus replication in various organs and to assess lung pathology (*21*).

On average, sCPD9-vaccinated hamsters gained slightly less weight than mock-vaccinated hamsters in the 21 days after vaccination, but differences were not significant (Fig. 1B). No adverse reactions or clinical signs, such as forced breathing or apathy, that would indicate severe disease were observed in any of the mock- or sCPD9-vaccinated hamsters. Attenuated sCPD9 virus replicated efficiently in the upper and lower respiratory tract of sCPD9-vaccinated hamsters on day 3 after vaccination (Fig. 1C). In addition, we detected infectious viruses in the lungs of two-thirds of sCPD9-vaccinated animals on day 3 after vaccination (Fig. 1C). Histopathological examination of lungs of sCPD9-vaccinated hamsters on day 3 after vaccination showed that sCPD9 is strongly attenuated, as only few mild inflammatory lesions could be detected in the lungs of vaccinated animals (*21*).

### Challenge infection

Vaccination with the sCPD9 virus induced strong protective immunity against challenge with all three SARS-CoV-2 variants. None of the vaccinated hamsters showed clinical signs of disease, and none of the challenged groups lost weight after challenge (Fig. 1D). In contrast, hamsters of the mock-vaccinated group showed typical signs of severe COVID-19–like disease, began to lose weight on day 2 after challenge, and continued to lose weight until the end of the study (day 5 after challenge) (Fig. 1D).

Virological assessments of the challenge infection confirmed virus replication in the upper airways of infected animals on days 2, 3, and 5 after challenge (Fig. 1E). The detection of viral RNA in the upper respiratory tract of many vaccinated animals suggests that the virus remains capable of establishing local infection, regardless of the variant used for challenge. Nevertheless, vaccination drastically reduced the amount of viral genomic RNA in the lower respiratory tract of challenge-infected hamsters (Fig. 1F). Vaccinated hamsters had distinctly lower viral loads than control animals on all days examined. The viral load in the control group was on average one- to sixfold higher on day 2 and four- to fivefold higher on days 3 and 5 after challenge (Fig. 1F). As expected, variants replicated with unequal efficiency in the lungs of challenged hamsters. Hamsters infected with the ancestral SARS-CoV-2 variant B.1, which is antigenically identical with the sCPD9 virus, produced the lowest viral loads, whereas those infected with B.1.351 virus had the highest viral loads.

To further evaluate the immunity established by vaccination, we attempted to isolate the challenge virus from lungs of animals in cultured Vero E6 cells. This evaluation showed sterilizing immunity in the lower airways of all but one subject. While we failed to isolate B.1 and B.1.1.7 viruses, we detected infectious, replication-competent virus in a single hamster (one of three hamsters) in the B.1.351 group, but only shortly after challenge infection, on day 2 after challenge (Fig. 1G).

### Histopathology after challenge

**Fig. 2.**
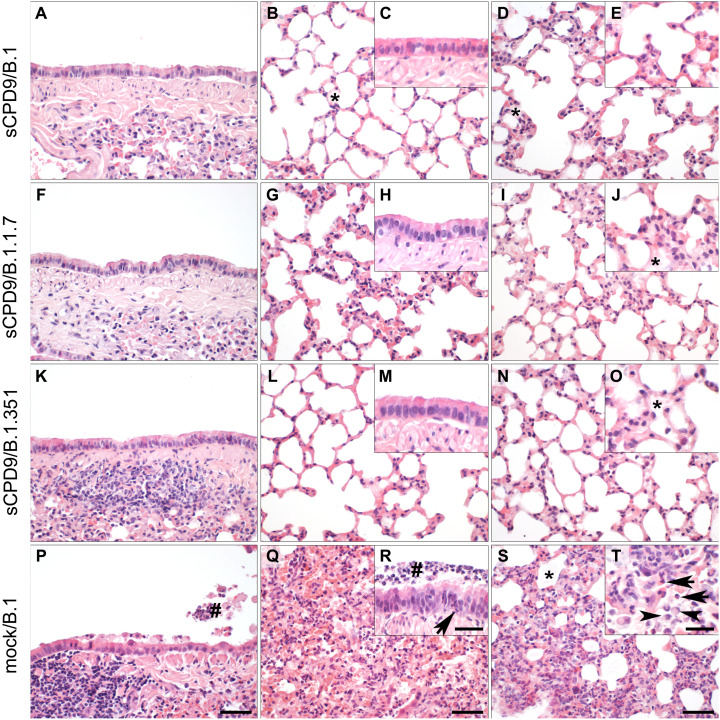
Pulmonary histopathology of mock/sCPD9-vaccinated and challenged Roborovski dwarf hamsters on days 2 (left column), 3 (middle column), or 5 (right column) after challenge. (**A** to **T**) Representative photomicrographs of hematoxylin and eosin (H&E)–stained, formalin-fixed, and paraffin-embedded lung tissue of infected hamsters. sCPD9-vaccinated hamsters were challenged with B.1, B.1.1.7, or B.1.351 virus on day 21 after vaccination. The lungs of sCPD9-vaccinated hamsters that were challenge-infected with three different SARS-CoV-2 variants showed very similar pulmonary morphology. Bronchioles had regular, columnar bronchiolar epithelium on days 2 and 3 after challenge (A, C, F, H, K, and M). Alveoli on days 3 (B, G, and L) and 5 after challenge (D, E, I, J, N, and O) showed mild to moderate infiltration by macrophages and few neutrophils with scattered mild alveolar edema (asterisk). In contrast, mock-vaccinated hamsters challenge-infected with the ancestral SARS-CoV-2 variant B.1 displayed typical lesions of COVID-19 at all times tested (P to T). After 2 days, the bronchiolar epithelium was flattened due to necrosis of bronchiolar epithelial cells and necrotic cellular debris, and degenerate neutrophils and macrophages were present in the bronchiolar lumen (hash) (P). In the course of disease, there was moderate to severe bronchiointerstitial pneumonia with necrosis of alveolar epithelial cells and massive infiltration by macrophages and neutrophils in the alveolar septa and alveolar spaces (Q). Similarly, bronchiolar epithelium was infiltrated by an increasing number of neutrophils (arrow) intraluminal cellular debris was accumulating (hash) (R). On day 5 after challenge, lungs showed predominantly interstitial pneumonia with remnants of alveolar edema fluid (asterisk) (S), neutrophils (T, arrow), and an increasing number of macrophages with prominently foamy cytoplasm (T, arrowhead). Scale bars, 50 μm (A, B, D, F, G, I, K, L, N, P, Q, and S) and 20 μm (C, E, H, J, M, O, R, and T).

To determine the protective capacity of vaccination with the sCPD9 virus against COVID-19–like disease caused by three different SARS-CoV-2 variants, the lungs of challenged hamsters were subjected to detailed histopathological examination (Fig. 2). The examination revealed that all sCPD9-vaccinated hamsters were fully protected against SARS-CoV-2–induced inflammatory damage to the lung, irrespective of the variant used for challenge infection (Fig. 2). In contrast, mock-vaccinated and B.1-infected hamsters developed severe lung pathology typical of experimental SARS-CoV-2 infection (*27*). These observations support the conclusion that vaccination with sCPD9 virus protected hamsters from major tissue damage and provided complete protection against COVID-19–like disease after challenge infection with three different SARS-CoV-2 variants.

### Serum neutralization after challenge infection

**Fig. 3.**
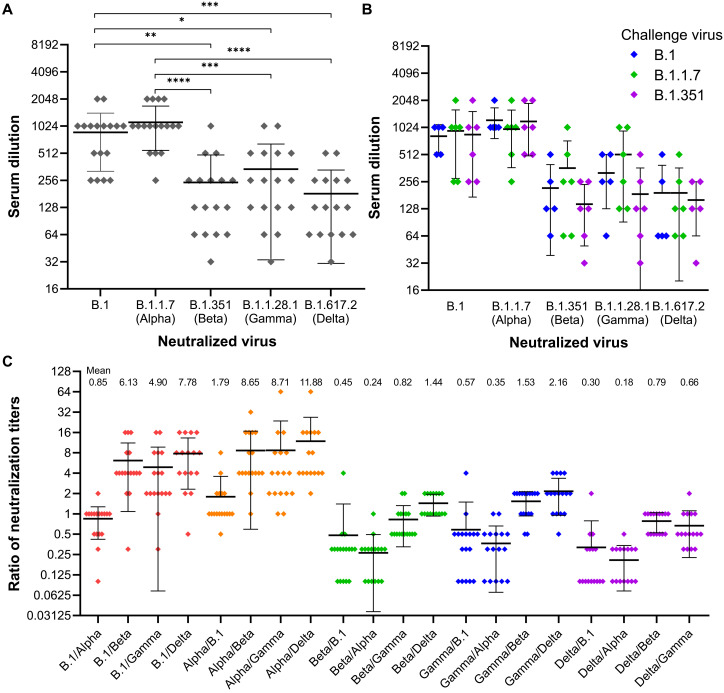
Sensitivity of SARS-CoV-2 variants B.1, B.1.1.7 (Alpha), B.1.351 (Beta), B.1.128.1 (Gamma), and B.1.617.2 (Delta) to neutralization by antibodies in sera of sCPD9-vaccinated Roborovski dwarf hamsters. (**A**) Neutralizing antibody titers of hamster sera collected on days 2 and 3 after challenge. Geometric mean and SD of the respective group titers are shown. The Kruskal-Wallis test with Dunn’s post hoc test showed that hamster sera neutralized the virus variants B.1.351, B.1.128.1, and B.1.617.2 significantly less efficiently than variants B.1 or B.1.1.7 (*P = 0.0264, **P = 0.0012, *** *P* < 0.001, and ****P < 0.0001). (**B**) Titers of neutralizing antibodies are plotted as a function of challenge virus. (**C**) Ratios of serum neutralization titers against different virus variants. The ratios show to which extent each hamster serum neutralizes different virus variants. The ratios of the neutralization titers of individual sera, the geometric mean, and the SD are shown. The geometric mean is also displayed as a number in the upper part of the diagram.

We quantified the capacity of 17 hamster sera that were collected on days 2 and 3 after challenge to neutralize SARS-CoV-2 variants B.1, B.1.1.7, B.1.351, B.1.128.1, and B.1.617.2 by a microneutralization assay (Fig. 3A). Sera from mock-vaccinated hamsters had no neutralization activity against all SARS-CoV-2 variants tested. Sera from sCPD9-vaccianted hamsters had strong neutralizing activity against the B.1 and B.1.1.7 variants, with geometric mean titers of 873 and 1129, respectively, but lower neutralizing activity against the B.1.351, B.1.128.1, or B.1.617.2 variants, with geometric mean titers of 243, 341, and 182, respectively (Fig. 3A). Challenge infection is expected to boost vaccine-induced immune responses and lead to production of antibodies that better neutralize antigens of the respective challenge SARS-CoV-2 variants. Because we collected sera shortly after the challenge, we did not expect to observe the induction of a variant-specific humoral response. Nevertheless, to account for possible challenge-induced effects, we also compared serum neutralization activity as a function of the challenge virus (Fig. 3B). This comparison showed that none of the different challenge viruses had any discernible effect on the neutralizing activity of the hamster sera. In other words, hamsters challenged with one variant, for example, B.1.351, did not produce a better humoral response against the same variant within 3 days after challenge. Furthermore, there was no significant difference in the neutralizing activity of sera collected on day 2 or 3 after challenge (fig. S1).

Vaccination with sCPD9 induced high levels of neutralizing antibodies that were similarly effective against not only the ancestral B.1 variant, which is antigenically identical with the sCPD9 virus, but also the evolutionarily more distant variant B.1.1.7, which has 17 mutations that alter or delete amino acids (8 mutations in the spike protein) compared with the original virus. Hamster sera also effectively neutralized the B.1.351 variant, albeit with six- to ninefold reduced efficacy (Fig. 3C). The results are in good agreement with previous studies, which showed that B.1.351 markedly decreased the neutralization activity of convalescent plasma of individuals that were infected with early SARS-CoV-2 lineages (~9- to 15-fold reduction) (*28*–*30*) and sera from individuals who have been vaccinated with vaccines containing the original version of spike protein (~10- to 14-fold reduction) (*30*, *31*).

Moreover, sera from vaccinated hamsters collected on days 2 and 3 after challenge also efficiently neutralized SARS-CoV-2 variants B.1.128.1 and B.1.617.2 (Fig. 3, A to C). These two variants were not used for challenge infection in this study, but were still neutralized with similar efficacy (~5- to 8-fold reduction in comparison to B.1) as the B.1.351 variant (~6-fold reduction). This result confirms that vaccination with the vaccine candidate sCPD9 elicits broad and potent neutralizing antibody responses against a wide variety of SARS-CoV-2 variants. Our data also show that among the virus variants studied here, variant B.1.617.2 is the most resistant to neutralization with antibodies that were induced by vaccination with a virus that contained the ancestral form of the spike protein. Recent studies have yielded similar results, showing that sera from vaccinated individuals and convalescent sera from individuals previously infected with variants other than B.1.617.2 neutralized the B.1.617.2 variant with a three- to sixfold reduced efficacy (*32*, *33*).

## DISCUSSION

Emerging variants are a constantly growing threat, because they not only dampen the efficacy of the licensed vaccines that target the original SARS-CoV-2 but also raise concerns that the virus may soon become resistant to current vaccines (*34*). Mutations in the spike protein have been shown to be primarily responsible for decreased neutralization of B.1.351 virus because they interfere with the efficient binding of neutralizing antibodies. Of particular importance is the E484K mutation in the receptor-binding domain of the spike protein, which prevents binding of the most potent neutralizing antibodies found in convalescent and postvaccination sera (*35*). This mutation is also present in B.1.1.28.1 and B.1.525 lineages and was also identified in some viruses of the B.1.1.7 and B.1.526 lineages.

Recent evidence has shown that some of the acquired mutations in the spike protein partially or severely compromise the efficacy of licensed vaccines that are based solely on said spike protein. For example, whereas Pfizer-BioNTech’s vaccine (BNT162b2, or Comirnaty) prevents 75% of mild and 90% of severe disease cases caused by the B.1.351 variant (*16*), the efficacy of Johnson & Johnson’s vaccine (Ad26.COV2.S) is 57 and 100%, respectively (*18*), and the efficacy of Novavax vaccine (NVX-CoV2373) is 49 and 100%, respectively (*36*). AstraZeneca’s vaccine (Vaxzevria or Covishield) has the lowest efficacy of all licensed vaccines, preventing only 10% infection with the variant B.1.351 (*17*). Similarly, although inactivated virus vaccines contain the entire antigenic repertoire of the virus and should theoretically provide better efficacy against emerging SARS-CoV-2 variants, in practice they offer the lowest efficacy against early virus variants among all licensed vaccines and therefore can offer only partial protection against emerging variants. For example, in Brazil, where 75% of infections were caused by variant B.1.1.28.1, the efficacy of Sinovac’s vaccine (CoronaVac) was only 50% in preventing symptomatic infection (*37*).

In the context of continuous SARS-CoV-2 evolution, our study yielded several important findings. We show that single-dose intranasal vaccination with a live attenuated virus vaccine candidate, sCPD9, induced complete protection against clinical disease and lung pathology caused by three different SARS-CoV-2 variants in a highly sensitive animal model. The challenge-infected hamsters did not develop any signs of disease and mounted strong neutralizing antibody responses. We show that B.1.351 virus, although markedly more resistant to neutralization by serum antibodies than B.1 or B.1.1.7 variants, was well controlled by vaccination with sCPD9. The fact that the lungs of B.1.351-challenged hamsters were completely protected from any damage suggests that vaccination with sCPD9 induced effective immune responses and thus compensated the reduced humoral immunity against variant B.1.351. Serum from vaccinated and challenged hamsters also showed neutralizing activity against B.1.1.28.1 and B.1.617.2, with results similar to what was determined for B.1.351. This finding suggests a protective effect of vaccination with sCPD9 against a wide variety of SARS-CoV-2 variants.

Safe blood collection from dwarf hamsters before euthanasia does not yield sufficient serum to determine serum neutralization titers before challenge infection. The lack of access to the sera limits our ability to assess the antibody responses by the vaccine in the absence of infection. Excellent clinical protection, consistent with the absence of replication-competent virus in the lungs of challenged animals, and robust antibody responses shortly after challenge do, however, demonstrate the strong protective potential of this vaccine against different SARS-CoV-2 variants. In addition, vaccination with a live attenuated virus may offer better protection from (re)infection than vaccines that carry only a single antigen, because all SARS-CoV-2 proteins may trigger stronger cytotoxic T cell responses (*38*).

While other studies have shown that emerging variants threaten the success of vaccination and rekindle the pandemic by diminishing the efficacy of licensed vaccines, our study shows that a single-dose intranasal vaccination with the lead live attenuated virus candidate sCPD9 induces immunity that provides excellent protection against currently circulating VOCs. It is conceivable that live attenuated virus vaccines, once fully developed, may offer a more practical solution on how to achieve protective immunity against such variants, instead of developing a specialized vaccine against each new dangerous variant that emerges. A live attenuated vaccine as described here may be particularly beneficial to boost and broaden immune responses after primary immunization with other vaccines. It remains to be determined to what extent the vaccination with the sCPD9 virus can protect against emerging VOCs in a nonhuman primate model or in humans.

## MATERIALS AND METHODS

### Cells and viruses

Vero E6 cells (American Type Culture Collection, CRL-1586) were grown in minimal essential medium (MEM) containing 10% fetal bovine serum (PAN Biotech), penicillin G (100 IU/ml), and streptomycin (100 μg/ml; Carl Roth) at 37°C and 5% CO_2_. SARS-CoV-2 variants B.1 (BetaCoV/Munich/BavPat1/2020) (*39*), B.1.1.7 (BetaCoV/Germany/ChVir21652/2020), B.1.351 (hCoV-19/Netherlands/NoordHolland_20159/2021), B.1.1.28.1 (hCoV-19/Netherlands/NoordHolland_10915/2021), and B.1.617.2 [SARS-CoV-2, Human, 2021, Germany ex India, 20A/452R (B.1.617)] were grown in Vero E6 cells. Virus stocks were stored at −80°C before experimental infections.

### Production of high-titer virus stocks of sCPD9

To produce high-titer virus stocks, confluent monolayers of Vero E6 cells grown in T-75 flasks were infected with sCPD9 virus at a multiplicity of infection of 1. Infected cells were cultured in 7 ml of cell culture medium and harvested at the onset of cytopathic effect on days 2 to 3 after infection. The virus stocks prepared in this manner typically contain approximately 1 × 10^7^ infectious virus particles per milliliter of cell culture.

### Virus titrations

The concentration of infectious viruses in virus stocks and in the lungs of infected animals was determined by titration on Vero E6 cells grown in 12-well plates. Stock titers of WT SARS-CoV-2 variants were determined by a plaque assay. Because sCPD9 virus does not produce readily visible plaques on Vero E6 cells, the titers of sCPD9 virus stocks were determined by a focus-forming assay. Briefly, Vero E6 cells were infected with 100 μl of serial 10-fold dilutions of virus. After 2 hours of incubation, the viral inoculum was removed and cells were overlaid with Dulbecco’s modified Eagle’s medium (DMEM) containing 2.5% microcrystalline cellulose (Avicel, FMC BioPolymer). Seventy-two hours after infection, cells were fixed with 4% formaldehyde, permeabilized with 0.1% Triton X-100, and blocked with 3% bovine serum albumin in phosphate-buffered saline (PBS). Cells were then incubated with a monoclonal mouse anti–SARS-CoV-2 nucleocapsid antibody (provided by S. Reiche, Friedrich Loeffler Institute, Riems, Germany) for 1 hour and then with goat anti-mouse immunoglobulin G–Alexa Fluor 568 secondary antibody (Thermo Fisher Scientific) for 45 min. To determine virus titers in the lung, 50 mg of lung tissue was first homogenized with a bead mill (Analytic Jena), the homogenate was serially diluted, and the virus titer was determined by focus-forming assay as described above.

After challenge infection of hamsters, virus titers in the lung of challenged animals were determined by plating 10-fold serial dilutions of 50 mg of lung homogenates onto Vero E6 cells. After incubation at 37°C for 2 hours, the homogenized tissue was aspirated, and cells were washed with PBS and overlaid with 2.5% microcrystalline cellulose (Avicel, FMC BioPolymer). Seventy-two hours after infection, cells were fixed with 4% formaldehyde and stained with crystal violet (0.75% aqueous solution), and plaques were counted.

### Ethics statement

In vitro and animal work was done under biosafety conditions in the biosafety level-3 (BSL-3) facility at the Institut für Virologie, Freie Universität Berlin, Germany. All animal experiments were approved by the Landesamt für Gesundheit und Soziales in Berlin, Germany (permit number 0086/20) and performed in compliance with relevant institutional, national, and international guidelines for care and humane use of animal subjects.

### Animal husbandry

The animal experiments were done in a certified BSL-3 facility. Five- to seven-week-old Roborovski dwarf hamsters (*P. roborovskii*) were purchased through the German pet trade from a single breeding facility. They were housed in groups of six animals in individually ventilated cages. The animals had unrestricted access to food and water and were allowed to acclimate to the conditions for 7 days before infection. During both experiments, cage temperatures ranged from 22° to 24°C and relative humidity ranged from 40 to 55%.

### Vaccination and challenge experiments

To assess the vaccine efficacy of the lead attenuated virus vaccine candidate sCPD9 in the Roborovski dwarf hamster, animals were randomly assigned into two groups, with 60% of the animals in each group being female. Twelve hamsters were mock-vaccinated with cell culture medium, and 30 hamsters were vaccinated with 1 × 10^5^ FFU of the vaccine candidate sCPD9 virus in 20 μl by intranasal instillation under anesthesia (*40*). Twenty-one days after vaccination, hamsters were challenge-infected with 1 × 10^5^ PFUs of challenge virus in 20 μl by intranasal instillation. Nine mock-vaccinated hamsters were challenged with the SARS-CoV-2 variant B.1, and nine sCPD9-vaccinated hamsters were challenged with either the SARS-CoV-2 variant B.1, B.1.1.7, or B.1.351. During the experiment, all hamsters were monitored twice daily for the clinical signs of disease and body weights were recorded. On day 3 after vaccination, and on days 2, 3, and 5 days after challenge (days 23, 24, and 26 of the experiment), three hamsters of each group were euthanized. Blood, tracheal swabs, and lungs were collected to determine virological and histological parameters of vaccination or infection. The left lungs were preserved in 4% formalin for subsequent histopathological detailed investigations.

### Histopathology and in situ hybridization

For histopathology, the left lung lobe was carefully removed and immersion-fixed in buffered 4% formalin, pH 7.0, for 48 hours. Lungs were embedded in paraffin and cut at 2 μm thickness. Sections were stained with hematoxylin and eosin (H&E) and periodic acid–Schiff (PAS) reaction followed by blinded microscopic evaluation by board-certified veterinary pathologist (A.D.G.) (*41*). H&E- and PAS-stained and in situ hybridization slides were analyzed and photographed using an Olympus BX41 microscope with the DP80 Microscope Digital Camera and the cellSens Imaging Software, version 1.18 (Olympus Corporation). For overviews with lower magnification, slides were automatically digitized using an Aperio CS2 slide scanner (Leica Biosystems), and image files were generated using the Image Scope Software (Leica Biosystems).

### RNA isolation and reverse transcription quantitative polymerase chain reaction

RNA was extracted from 25 mg of lung homogenates and tracheal swabs using the innuPREP Virus RNA Kit (Analytik Jena). SARS-CoV-2 RNA copies were quantified using the NEB Luna Universal Probe One-Step RT-qPCR Kit (New England Biolabs) on the StepOnePlus Real-Time PCR System (Thermo Fisher Scientific) as previously described (*42*).

### Serum virus neutralization assay

Microneutralization assay was done with six sera from hamsters that were mock-vaccinated and challenge-infected with the B.1 virus variant, and with five, six, and six sera from hamsters that were sCPD9-vaccinated and challenge-infected with the B.1, B.1.1.7, or B.1.351 virus variants, respectively. Each serum was tested for neutralization of the B.1, B.1.1.7, B.1.351, B.1.1.28.1, or B.1.617.2 variant. Microneutralization assay was performed by twofold serial dilutions (1:16 to 1:2048) of complement-inactivated (56°C, 30 min) hamster serum plated on subconfluent monolayers of Vero E6 cells in 96-well plates. Cells in each well were infected with 100 PFUs of the respective SARS-CoV-2 variant, incubated for 72 hours at 37°C, fixed with 4% formalin for 24 hours, and stained with crystal violet (0.75% aqueous solution). Serum neutralization was considered effective in wells that showed no any cytopathic effect, and the last effective dilution was counted.

## Notes

View/request a protocol for this paper from *Bio-protocol*.
