# Supplementary material for: Live attenuated virus vaccine protects against SARS-CoV-2 variants of concern B.1.1.7 (Alpha) and B.1.351 (Beta)
Source: Sci Adv. 2021 Dec 1;7(49):eabk0172. doi: 10.1126/sciadv.abk0172 (PMC8635430; doi:10.1126/sciadv.abk0172)
Supplement: Supplementary file 1 — Fig. S1 [file sciadv.abk0172_sm.pdf]

Science Advances
AAAS

Supplementary Materials for

Live attenuated virus vaccine protects against SARS-CoV-2 variants of concern B.1.1.7 (Alpha) and B.1.351 (Beta)

Jakob Trimpert, Julia M. Adler, Kathrin Eschke, Azza Abdelgawad, Theresa C. Firsching, Nadine Ebert, Tran Thi Nhu Thao, Achim D. Gruber, Volker Thiel, Nikolaus Osterrieder, Dusan Kunec*

*Corresponding author. Email: dusan.kunec@fu-berlin.de

Published 1 December 2021, *Sci. Adv.* **7**, eabk0172 (2021)
DOI: 10.1126/sciadv.abk0172

This PDF file includes:

Fig. S1

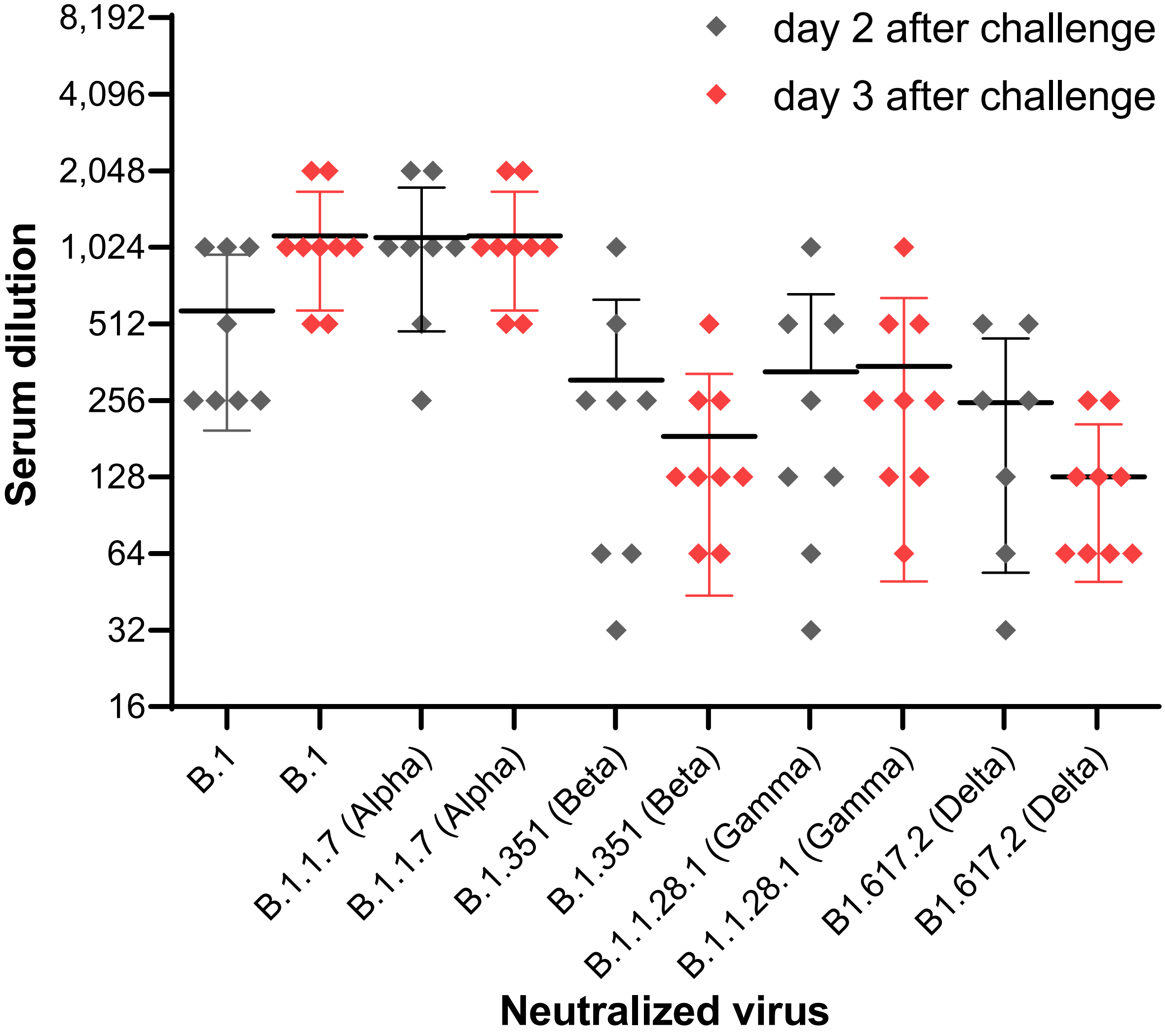

Fig. S1.

Sensitivity of SARS-CoV-2 variants B.1, B.1.1.7 (Alpha), B.1.351 (Beta), B.1.128.1 (Gamma), and B.1.617.2 (Delta) to neutralization by antibodies in sera of sCPD9-vaccinated and challenged Roborovski dwarf hamsters collected on days 2 and 3 after challenge.
